# Cost-utility analysis of adjuvant goserelin (Zoladex) and adjuvant chemotherapy in premenopausal women with breast cancer

**DOI:** 10.1186/1471-2407-12-33

**Published:** 2012-01-21

**Authors:** Tsui Fen Cheng, Jung Der Wang, Wu Ching Uen

**Affiliations:** 1 Department of Surgery, Shin Kong Wu Ho-Su Memorial Hospital, 95 Wen Chang Road Shih Lin, Taipei, Taiwan; 2 Department of Public Health National Cheng Kung University College of Medicine and Hospital, Tainan, Taiwan; 3 Department of Medical Oncology, Shin Kong Wu Ho-Su Memorial Hospital, 95 Wen Chang Road Shih Lin Taipei, Taiwan

**Keywords:** Adjuvant chemotherapy, Adjuvant hormone therapy, Goserelin, Estrogen-responsive (ER+), EORTC-QLQ-C30, QALY, Breast cancer

## Abstract

**Background:**

Increased health care costs have made it incumbent on health-care facilities and physicians to demonstrate both clinical and cost efficacy when recommending treatments. Though studies have examined the cost-effectiveness of adjuvant goserelin with radiotherapy for locally advanced prostate cancer, few have compared the cost-effectiveness of adjuvant goserelin to adjuvant chemotherapy alone in premenopausal breast cancer.

**Methods:**

In this retrospective study at one hospital, the records of 152 patients with stage Ia to IIIa ER + breast cancer who received goserelin or chemotherapy were reviewed. Survival analysis was assessed by the Kaplan-Meier method. Patients were interviewed to evaluate their quality of life using the European Organization for Research and Treatment Quality of Life questionnaire (EORTC-QLQ-C30, version 4.0), and to obtain the utility value by the standard gamble (SG) and visual scale (VS) methods. Total medical cost was assessed from the (National Health Insurance) NHI payer's perspective.

**Results:**

Survival at 11 years was significantly better in the groserelin group (*P *< 0.0012). The lifetime lost was lower in the goserelin group (42 months vs. 66 months). The quality adjusted survival (QAS) of patients who received goserelin was longer (122.5 ± 6.3 vs. 112.2 ± 6.7 months). Total expenses of goserelin were more than cyclophosphamide, methotrexate, 5-fluorouracil (CMF) or 5-fluorouracil, epirubicin, cyclophosphamide (FEC) chemotherapy regimes, but less than docetaxel, epirubicin (TE) or docetaxel, epirubicin, cyclophosphamide (TEC) regimes. The quality-adjusted life-year was higher in the goserelin group.

**Conclusions:**

Goserelin therapy results in better survival and higher utility-weighted life-years, and is more cost-effective than TC or TEC chemotherapy.

## Background

Endocrine therapy for premenopausal patients with primary breast cancer is based on reducing circulating levels of estrogens. Because the ovary is a major contributor of circulatory estrogen, therapies revolve around reducing ovarian production. Chemotherapy decreases estrogen levels due to cytotoxic effects on the ovaries. Other available therapies to reduce estrogen levels include ovarian ablation by either surgical removal or radiotherapy, and pharmacological anti-estrogen therapy, primarily with tamoxifen. Nevertheless, for premenopausal women with breast cancer, protection of ovarian function is important in order to maintain quality of life (QoL) and fertility.

Luteinizing hormone-releasing hormone (LHRH) analogues provide an alternative therapy. LHRH analogues decrease ovarian estradiol production indirectly by acting on the hypothalamic-pituitary-ovarian axis and inhibiting the secretion of pituitary gonadotrophins [1]. Chronic and reversible suppression of gonadotropin secretion leads to a loss of ovarian steroid production--an optimal antitumor environment for estrogen-responsive (ER+) tumors. For younger, premenopausal ER + breast cancer patients, additional endocrine therapies are recommended in order to increase the effectiveness of the primary treatment [2].

Goserelin (Zoladex^®^; Astra-Zeneca Pharmaceuticals LP, Wilmington, DE, UK) is a widely-used LHRH analogue shown to be effective and well-tolerated in patients with advanced breast cancer, and has recently been the focus of an international study that investigated its effects on amenorrhea, hot flashes, and QoL as compared to chemotherapy, or chemotherapy followed by goserelin [3]. Since it has been observed to protect ovarian function from damage by chemotherapy, it is also used to decrease gonado-toxicity and prevent premature menopause induced by chemotherapy in young, early breast cancer patients when administered before and during treatment [4,5]. Goserelin is associated with fewer adverse events and comparative long-term disease-free survival and quality of life in patients with ER + breast cancer compared to chemotherapy alone [3,6-8].

Quality of life is usually measured with a utility scale or a health profile, and then summarized numerically. From this, the expected quality-adjusted survival (QAS) time can be calculated, which takes into account each of the patient's various health states and the survival duration (in years) spent in each state. The quality-adjusted life-year (QALY) is also used for outcome evaluation, i.e., for comparing the overall impact from both mortality and morbidity of different health-related events [9,10]. In this study, both the QAS and QALY of patients who received goserelin were estimated.

Another method for constructing a health profile for long-term cancer survivors is to use survival-weighted psychometric scores (SWPS) as an endpoint to compare the efficacy of cancer treatments. The SWPS calculation may also be extrapolated beyond the follow-up limit of the patient cohort to obtain a life-long estimation of QoL changes [11]. A semiparametric method can be used to estimate life expectancy (LE), and foretell the expected years of life lost (EYLL). The calculations can be applied to clinical trials and can also be merged with data pertaining to QoL, resulting in a more detailed outcome assessment, effective optimization of cancer management, best use of different treatment protocols, and most efficient resource allocation [12]. Because hormone suppression treatment with goserelin is reversible at the end of therapy, unlike oophorectomy and ablation, it is an ideal candidate for a detailed study that integrates QoL measurements.

Increased health care costs have made it incumbent on health-care facilities and physicians to demonstrate both clinical and cost efficacy when recommending treatments. Though studies have examined the cost-effectiveness of adjuvant goserelin with radiotherapy for locally advanced prostate cancer [13,14], few have compared the cost-effectiveness of adjuvant goserelin to adjuvant chemotherapy alone in premenopausal breast cancer patients. The present study evaluated the cost-effectiveness of adjuvant goserelin or adjuvant chemotherapy in stage Ia to IIIa ER + breast cancer patients using health related quality of life (HRQoL) data. Costs were estimated from the perspective of the National Health Insurance (NHI; a mandatory health insurance employed in Taiwan) payer.

## Methods

### Study design

Medical history and chart data of patients admitted to the Department of General Surgery, Shin Kong Wu Ho-Sun Memorial Hospital, from 1993 to 2007 were reviewed. Eligible patients fulfilled the following criteria: 1) diagnosed with breast cancer before menopause; 2) clinical staging between Ia and IIIa; 3) adjuvant chemotherapy (at least 6 cycles) or goserelin therapy. Patients who received tamoxifen were excluded from the analysis.

Patients either underwent goserelin therapy (3.6 mg subcutaneous depot injection into the abdominal wall every 4 weeks) or adjuvant chemotherapy (6 cycles of combined therapy of CMF [cyclophosphamide, methotrexate, 5-fluorouracil], FEC [5-fluorouracil, epirubicin, cyclophosphamide], TE [docetaxel, epirubicin], or TEC [docetaxel, epirubicin, cyclophosphamide]). Chemotherapy was dosed by square meter of body surface area (BSA); common BSA measurements for women are between 1.5 m^2 ^and 1.8 m^2^. Mean survival curves were calculated for the 2 treatment groups, and subsequently adjusted for QoL. Between August 30^th^, 2007 and December 29^th^, 2007 152 patients with stage Ia to IIIa disease who received goserelin for at least 1 year, or received at least 6 cycles of chemotherapy as adjuvant therapy were interviewed to evaluate their QoL using the European Organization for Research and Treatment QoL questionnaire (EORTC-QLQ-C30, version 4.0), and to obtain the utility value by the standard gamble (SG) and visual scale (VS) methods. All interviews were performed by 1 of 2 trained interviewers, and lasted on average for 60 min.

### Total medical cost

Total medical costs (surgical intervention, drugs, and other health care services) were assessed from a payer's perspective, and based on standard claims submitted to the NHI. Additional charges for blood tests, artificial vessel placement (for chemotherapy), and granulocyte colony-stimulating factor (GCSF) hormone, were also included. Costs were expressed in Taiwan New Dollars (TWD) and US dollars (USD), where 1 USD = 32 TWD.

### Quality of life and utility values

EORTC-QLQ-C30 is a reliable and sensitive 30-item, cancer-specific, self-administered structured questionnaire designed to assess the quality of life of cancer patients participating in international clinical trials [15]. All scales and single-item measures range in score from 0 to 100, with a higher score representing a higher response level, or healthier level of functioning. A high score on the global health status scale represents a high QoL, though a high score for a symptom scale/item represents a high level of symptoms/problems. A multi-attribute utility scoring formula, based on SG utilities derived from the power conversion of VS scores, was used to calculate a utility score that reflects a respondent's preferences for the assessment of his/her health status [16]. The multi-attribute utility analysis allows an assignment of values to an individual's health without having to employ costly valuation procedures.

### Statistical methods

EORTC-QLQ-C30 data were scored using the EORTC-QLQ-C30 scoring manual [17]. Reference data was matched for each patient, according to age and gender from the Life Table of Taiwan, 1994. Expected years of life lost were calculated by reference life year survival to subtract index life year survival [8]. An 11-year and 50-year survival analysis was performed by the Kaplan-Meier method. QAS was assessed by multiplying the overall survival for each year (determined by the area under the Kaplan-Meier curve) by the average EORTC-QLQ-CL30 score. To assess whether incremental costs of goserelin therapy or chemotherapy were justified from the perspective value attributed to different outcomes between the 2 treatment groups, the QALY was calculated in terms of gain. QALYs gained were calculated by: ICER = (cost*_goserelin _*- cost*_chemotherapy_*)/(QALY*_goserelin _*-QALY*_chemotherapy_*).

Survival times of up to 600 months (50 years) were calculated because the life expectancy of breast cancer is about 20 years, and some of the patients are relatively young (e.g., 25-40 years), thus there are patients who may survive more than 30 or 40 years [12]. Therefore, in order to accurately estimate the lifetime survival function, we extrapolated to 50 years. The method has been mathematically proven to be valid if the assumption of constant excess hazard holds [18]. In fact, such an assumption generally holds for most cancers causing premature mortality, namely, patients with breast cancer who survive generally share the same likelihood of dying of other common causes (acute myocardial infarction, stroke, traffic injuries, etc.) in addition to their breast cancer after the first 1-2 years of life. Otherwise, the survival ratio of breast cancer to age-, gender-matched referents would become stable after the first 1-2 years of diagnosis.

Data were analyzed using SAS 9.0 (SAS Institute Inc., Cary, NC, USA) and a value of P < 0.05 was considered statistically significant.

## Results

### Patients

A total of 564 patient charts were reviewed. Patients who underwent goserelin therapy presented with a younger mean age (40.8 ± 6.5 years), shorter follow-up duration (168 months vs. 273 months), and a higher 10-year survival rate (Table 1).

**Table 1:** Patient demographic data

	Adjuvant therapy	
	
	Goserelin	Chemotherapy
Age (year)	40.8 ± 6.5	41.9 ± 5.7
Follow-up (month)	168	273
10-year survival rate	0.88	0.82
Life expectancy of patient population (months)	432 ± 23	401 ± 17
Life expectancy of reference population (months)	474 ± 1	467 ± 1
Life time lost (months)	42	66

**Figure 1:**
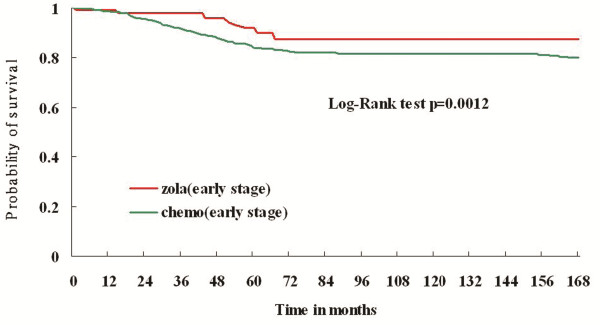
**Survival curves of adjuvant goserelin and adjuvant chemotherapy for premenopausal breast cancer patients**.

**Figure 2:**
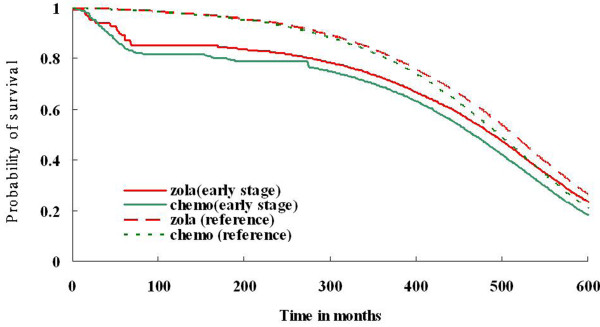
**Survival curves of adjuvant goserelin and adjuvant chemotherapy for premenopausal breast cancer patients with extrapolation of 50 years**.

From July 2007 to December 2007, 152 patients with stage Ia to IIIa disease who received goserelin for at least 1 year or received at least 6 cycles of chemotherapy as adjuvant therapy were interviewed to obtain the utility value by the SG and VS methods. The 11-year survival rate, described in Figure 1, indicates that patients who received goserelin had a significantly better survival rate than chemotherapy patients (P < 0.002). Figure 2, which presents the extrapolated 50-year survival rates, also reveals consistent findings.

### QoL and utility values

Results of the EORTC-QLQ-C30 questionnaire revealed that patients who received goserelin therapy had a higher SG utility score than those who received chemotherapy (0.81 ± 0.17 vs. 0.78 ± 0.23) (Table 2). Patients who received goserelin therapy also displayed a lesser amount of life-time lost (42 months vs. 66 months), and a longer life expectancy (432 ± 23 vs. 401 ± 17 months). A comparatively lower QAS (341 ± 31 vs. 336 ± 39) was observed for goserelin patients (Table 3).

**Table 2:** HRQoL utility values as per multi-attribute utility analysis

	Goserelin	Chemotherapy
Age	42.6 ± 7.3	45.6 ± 6.5
Mean of SG	0.81 ± 0.17	0.78 ± 0.23

**Table 3:** Quality-adjusted survival (QAS) estimated from a sample through the standard gamble (SG) method

	Goserelin	Chemotherapy
Life expectancy of patient population	432 ± 23	401 ± 17
Life expectancy of reference population	474 ± 1	467 ± 1
SG of patient population	0.81	0.78
SG of reference population	1	1
QAS of patient population	341 ± 31	336 ± 39
QAS of reference population	476 ± 1	468 ± 1
QAS lost	135	132

**Figure 3:**
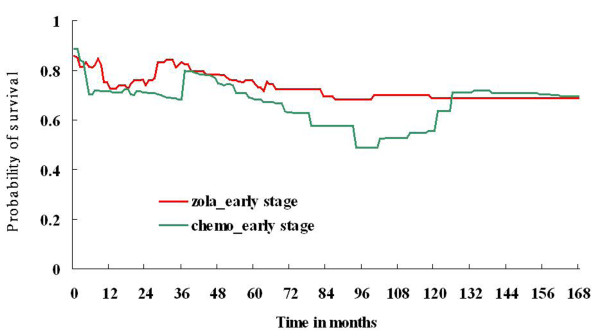
**Quality-adjusted survival (QAS) of adjuvant goserelin and adjuvant chemotherapy for premenopausal breast cancer patients**.

**Figure 4:**
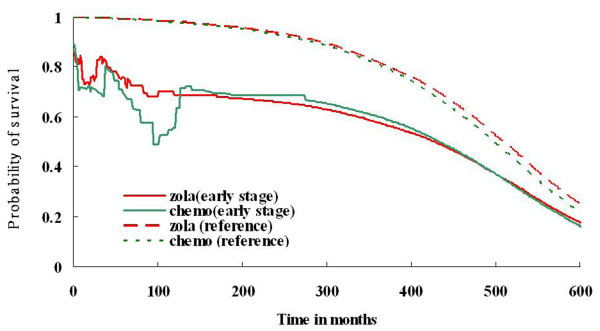
**Quality-adjusted survival (QAS) of adjuvant goserelin and adjuvant chemotherapy for premenopausal breast cancer patients with extrapolation of 50 years**.

Quality-adjusted survival (QAS) of adjuvant goserelin and adjuvant chemotherapy is shown in Figure 3. The QALE of patients who received goserelin at an early stage (122.5 ± 6.3 months) was longer than patients who received chemotherapy at an early stage (112.2 ± 6.7 months). Figure 4 presents the QAS of adjuvant goserelin and adjuvant chemotherapy for patients extrapolated to 50 years. The QALE of patients who received goserelin at an early stage (340.9 ± 30.6 months) was longer than for patients who received chemotherapy at an early stage (335.7 ± 39.2 months).

### Cost analysis

The total costs for 2 years of goserelin therapy and 6 cycles of chemotherapy from the perspective of the NHI payer are presented in Table 4. Based on a BSA of 1.5 m^2^, the total cost of goserelin (USD 5,532) was greater than that of CMF (USD 1,789) and FEC (USD 3,604), but less expensive than TE (USD 12,453) and TEC (USD 12,517). A similar result was found when the costs were calculated based on a BAS of 1.8 m^2^. The QALY was higher in the goserelin group as compared to chemotherapy groups by either SG or VS. Goserelin versus CMF had the highest ICERs among the 4 chemotherapeutic drug regimes. The ICERs were: 1,891 USD (BSA 1.5 m^2^) or 1,887 USD (BSA 1.8 m^2^) by SG, and 2,283 USD (BSA 1.5 m^2^) or 2,279 USD (BSA 1.8 m^2^) by VS. Goserelin versus TEC had the lowest ICERs:

**Table 4:** The total costs (USD) in the adjuvant treatment of premenopausal patients with stage Ia to IIIa breast cancer in Taiwan

	Drug Costs		Surgery	G-CSF Treatment	Blood Testing	Chemo Costs	Total Costs	
						
	BSA 1.5 m^2^	BSA 1.8 m^2^					BSA 1.5 m^2^	BSA 1.8 m^2^
Goserelin	5,532		0	0	0	0	5,532	

Chemotherapy								
CMF	149	156	438	651	59	492	1,789	1,795
FEC	1,964	1,970	438	651	59	492	3,603	3,610
TE	10,813	12,145	438	651	59	492	12,453	13,785
TEC	10,878	12,210	438	651	59	492	12,517	13,849

BSA, body surface area; CMF, (cyclophosphamide, methotrexate, 5-fluorouracil); FEC, (5-fluorouracil, epirubicin, cyclophosphamide); TE (docetaxel, epirubicin); TEC, (docetaxel, epirubicin, cyclophosphamide)

**Table 5:** ICER of goserelin vs. chemotherapy in the adjuvant treatment of breast cancer

	ΣQALY		ΣCost		ICER^a^ Goserelin vs. Chemotherapy			
	
	SG	VS	BSA 1.5 m^2^	BSA 1.8 m^2^	SG		VS	
					
					BSA 1.5 m^2^	BSA 1.8 m^2^	BSA 1.5 m^2^	BSA 1.8 m^2^
Goserelin	8.81	8.78	5,532					

Chemotherapy								
CMF	6.83	7.14	1,789	1,792	1,891	1,887	2,283	2,279
FEC	6.83	7.14	3,606	3,610	974	971	1,176	1,172
TE	6.83	7.14	12,453	13,785	-3,495	-4,168	-4,220	-5,032
TEC	6.83	7.14	12,517	13,849	-3,528	-4,200	-4,259	-5,071

^a^ICER = (cost*_goserelin _*- cost *_chemothreapy _*)/(QALY*_goserelin _*- QALY*_chemothre_)*

BSA, body surface area; CMF, (cyclophosphamide, methotrexate, 5-fluorouracil); FEC, (5-fluorouracil, epirubicin, cyclophosphamide); TE (docetaxel, epirubicin); TEC, (docetaxel, epirubicin, cyclophosphamide)

Cost in US dollars

-3,528 USD (BSA 1.5 m^2^) or -4,200 USD (BSA 1.8 m^2^) by SG, and -4,259 USD (BSA 1.5 m^2^) or -5,071 USD (BSA 1.8 m^2^) by VS.

## Discussion

There are approximately 6,500 new cases of breast cancer diagnosed per year in Taiwanese women, and most are younger in age and more likely to be premenopausal than their Western counterparts [19]. A recent report indicated that > 50% of the total breast cancer cases annually in Taiwan and China are in premenopausal patients [19]. In order to maintain QoL and fertility, protection of ovarian function is crucial for women undergoing breast cancer therapy.

LHRH analogues decrease ovarian estradiol production indirectly by impinging on the hypothalamic-pituitary-ovarian axis, and inhibiting secretion of pituitary gonadotrophins [2]. Chemotherapy usually results in gonado-toxicity and induces damage of ovarian function. Goserelin has been observed to protect ovarian function; therefore, it is unlikely to induce premature menopause and osteoporosis in young women [1,5]. In a phase II pilot study, the addition of goserelin to adjuvant therapy of premenopausal patients with early breast cancer was well tolerated and shown to protect ovarian function [3]. In our study, 86% of patients who were treated with goserelin resumed normal menses, and 1 patient had a pregnancy that ended with a normal childbirth 5 years after treatment.

The present study evaluated the cost-effectiveness of adjuvant goserelin therapy versus adjuvant chemotherapy in premenopausal breast cancer patients with stage Ia to IIIa disease. To our knowledge, this is the first study evaluating the integration of goserelin into adjuvant hormonal therapy in premenopausal breast cancer. An illuminating study exploring women's treatment preferences found that when healthy, premenopausal women were given the choice of adjuvant goserelin or CMF chemotherapy upon hypothetically developing ER + breast cancer, an overwhelming number chose goserelin over chemotherapy [20]. The primary reasons for choosing goserelin were to avoid the general side effects of chemotherapy, in particular hair loss, and a lesser disruption of the activities of normal life with goserelin as compared to chemotherapy. Other factors such as fertility, length of treatment, and amount of travel required to receive treatments were discussed in the study; however, no comments regarding the cost of treatment were made.

The results of our study indicate that goserelin is particularly cost-effective compared to TE and TEC chemotherapy regimens, and comparable to CMF and FEC. The cost of 2-year goserelin therapy appears more expensive ($5,273 USD) than both CMF (approximately $1,666 USD) and FEC (approximately $1,872), regardless of BSA, but higher QALY is seen. Based on the EORTC-QLQ-CL30 scores from our study, patients who receive goserelin therapy have higher QoL. Similarly, there are positive ICERs for goserelin vs. CMF and FEC, but negative ICERs for goserelin vs. TE and TEC.

There have been 4 large multi-center studies comparing efficacy outcomes between adjuvant goserelin (3.6 mg depot) and adjuvant chemotherapy. These are the German Adjuvant Breast Cancer Group (GABG) trial IV-A-93 [21], the Austrian Breast and Colorectal Cancer Study Group Trial 5 (ABCSG) [22], the International Breast Cancer Study Group (IBCSG) Trial VIII [7], and the Zoladex Early Breast Cancer Research Association Trialists' Group (ZEBRA) [6]. Of these, the IBCSG trial VIII, ABCSG trial VI, and ZEBRA examined CMF for 6 cycles (28 days each), whereas the GABG trial IV-A-93 examined intravenous cyclophosphamide.

The ZEBRA study [6], the largest, consisted of primarily ER + patients (1189/1614, 73.7%,) and demonstrated comparable recurrence-free survival, overall survival, and frequency of adverse effects at 6 years for ER + patients, but not for ER- and ER-unknown status patients, a trend seen in the other trials. The IBCSG and ZEBRA trials also indicated significantly better QoL during the first year in patients receiving goserelin, but little difference thereafter [6,23]. The ABCSG trial concluded that goserelin and tamoxifen were significantly more effective, with increased local recurrence-free survival and relapse-free survival as compared to CMF premenopausal women with stage I and II breast cancer [22]. An update on the ZEBRA study at a median follow-up of 7.3 years confirmed the previously reported outcomes for overall survival, and demonstrated the effectiveness of goserelin as an alternative to CMF for adjuvant therapy of premenopausal ER + women with early breast cancer [24].

Taken together, the results of the aforementioned trials are consistent with the present finding that adjuvant CMF and FEC produced comparative HRQoL utility values (0.81 vs. 0.78) at last follow-up, and appeared more economically sound than goserelin, in premenopausal patients with early stage breast cancer. It should be noted, however, that although the cost of goserelin may seem exorbitant relative to CMF and FEC, hormonal therapy administered in the early stages of breast cancer is likely to be economical when considering the outcomes of other common medical interventions.

While no cost-utility studies exist on adjuvant goserelin therapy in breast cancer alone, analyses of CMF and FEC have been extensively reported. Total costs of adjuvant CMF therapy in breast cancer have been reported to be approximately $3,852 USD-$10,197 USD (for 9 cycles) in Norway 1998-2000 [25], which amounts to approximately $1,688 USD per life-year saved in the US in the year 1992 [26], with drug costs accounting for 40% in both cases. CMF (for 6 cycles) in the present study is the most affordable regimen (approximately $1,788 USD for a BSA of 1.5 m^2 ^and $1,795 USD for a BSA of 1.8 m^2^), and differences in costs may likely reflect differences in national costs, rather than any major differences in treatment regimen.

Our review of prior economic evaluation research related to breast cancer treatment revealed the first cost-benefit analysis report discussing post-surgery adjuvant therapy for breast cancer was published in the early 1990s [27]. In the evaluation report comparing tamoxifen, chemotherapy, and combination therapy, Smith analyzed treatments that were suitable for different disease symptoms in patients with breast cancer > 45 years old and before menopause. The authors concluded that in premenopausal early-stage breast cancer, chemotherapy adds substantial clinical benefit at a modest cost while tamoxifen alone adds meaningful benefit only in ER + cancer, and that combined therapy is effective for all women, but is most beneficial and only cost-effective in ER + women.

Several reports evaluating the cost-benefit of chemotherapy for breast cancer exist in the literature. One report analyzing node positive breast cancer patients and comparing the cost-benefit ratio with/without CMF as adjuvant therapy revealed an ICER value of approximately $447 USD per person, per year [28]. Another study comparing the cost-benefit of 2 combination therapies, TAC (docetaxel, doxorubicin, and cyclophosphamide) and FAC (fluorouracil, doxorubicin, and cyclophosphamide), utilized a decision-making model from the England National Health Service [29]. The result showed that when using FAC as a standard, for an increase of 1 unit of TAC per person, per year, the corresponding cost was £15,418, while for an increase of 1 unit of QALY, the corresponding cost was £18,188.

The present study has a number of limitations. First, using multi-attribute utility analysis may have resulted in an overestimated VS before power conversion into an SG score. Also, robustness of the utility analysis could not be ascertained, as a sensitivity analysis was not performed. Second, the results reflect the NHI system of Taiwan. Thus, when estimating costs, total medical costs (surgical intervention, drugs, and other health care services) were assessed from a payer's perspective, and based on standard claims submitted to the NHI. However, the NHI has restrictions on the prescribing of goserelin. Prescription of goserelin requires pre-registry, and is limited to patients who are unsuitable for hysterectomy, or fail to respond to other hormone therapy (e.g., tamoxifen, megestrol). Therefore, goserelin is considered a second line therapeutic agent. Additionally, the cost data that was utilized reflects the Taiwanese societal perspective. Thus, cost-utility thresholds employed in other countries cannot be directly applied. Lastly, because the data retrieved was from the time period of 1993 to 2007, there was a large amount of missing data with respect to patient characteristics, thus we only presented the data set that was complete, age.

## Conclusion

In conclusion, this study indicates that adjuvant goserelin therapy in premenopausal women with breast cancer is particularly cost-effective when compared to TE and TEC adjuvant chemotherapy regimens, but more expensive (at the cost of higher QALY gained) when compared to CMF and FEC. Both goserelin and chemotherapy demonstrate comparable efficacy in terms of HRQoL at final follow-up. Goserelin, however, leads to a better QoL for younger breast cancer patients.

## Abbreviations

BSA: body surface area; CMF: cyclophosphamide methotrexate, 5-fluorouracil; EORTC-QLQ-C30: European Organization for Research and Treatment quality of life questionnaire; FAC: fluorouracil doxorubicin, cyclophosphamide; FEC: 5-fluorouracil epirubicin, cyclophosphamide; HRQoL: health related quality of life; ICER: incremental cost-effectiveness ratio; QALY: quality-adjusted life-year; QAS: quality-adjusted survival; TAC: docetaxel doxorubicin, cyclophosphamide; TE: docetaxel epirubicin; TEC: docetaxel epirubicin, cyclophosphamide.

## Competing interests

The authors declare that they have no competing interests.

## Author details

Breast specialist, Department of General Surgery, Shin Kong Wu Ho-Su Memorial Hospital N0 .95 Wen Chang Road, Shih Lin, Taipei, Taiwan.

## Authors' contributions

TF participated in the design of the study, the assemblage and the statistical analysis of the data and the drafting of the manuscript. JD participated in the design and calculation of the method of extrapolation, WC participated in chemotherapy and the collection of the data. All authors read and approved the final manuscript.

## Pre-publication history

The pre-publication history for this paper can be accessed here:

http://www.biomedcentral.com/1471-2407/12/33/prepub
